# Beneficial effects of elafibranor on NASH in E3L.CETP mice and differences between mice and men

**DOI:** 10.1038/s41598-021-83974-8

**Published:** 2021-03-03

**Authors:** Anita M. van den Hoek, Lars Verschuren, Martien P. M. Caspers, Nicole Worms, Aswin L. Menke, Hans M. G. Princen

**Affiliations:** 1 Department of Metabolic Health Research, The Netherlands Organization for Applied Scientific Research (TNO), Leiden, The Netherlands; 2 Department of Microbiology and Systems Biology, The Netherlands Organization for Applied Scientific Research (TNO), Zeist, The Netherlands

**Keywords:** Metabolic syndrome, Pharmaceutics, Preclinical research, Experimental models of disease

## Abstract

Non-alcoholic steatohepatitis (NASH) is the most rapidly growing liver disease that is nevertheless without approved pharmacological treatment. Despite great effort in developing novel NASH therapeutics, many have failed in clinical trials. This has raised questions on the adequacy of preclinical models. Elafibranor is one of the drugs currently in late stage development which had mixed results for phase 2/interim phase 3 trials. In the current study we investigated the response of elafibranor in APOE*3Leiden.CETP mice, a translational animal model that displays histopathological characteristics of NASH in the context of obesity, insulin resistance and hyperlipidemia. To induce NASH, mice were fed a high fat and cholesterol (HFC) diet for 15 weeks (HFC reference group) or 25 weeks (HFC control group) or the HFC diet supplemented with elafibranor (15 mg/kg/d) from week 15–25 (elafibranor group). The effects on plasma parameters and NASH histopathology were assessed and hepatic transcriptome analysis was used to investigate the underlying pathways affected by elafibranor. Elafibranor treatment significantly reduced steatosis and hepatic inflammation and precluded the progression of fibrosis. The underlying disease pathways of the model were compared with those of NASH patients and illustrated substantial similarity with molecular pathways involved, with 87% recapitulation of human pathways in mice. We compared the response of elafibranor in the mice to the response in human patients and discuss potential pitfalls when translating preclinical results of novel NASH therapeutics to human patients. When taking into account that due to species differences the response to some targets, like PPAR-α, may be overrepresented in animal models, we conclude that elafibranor may be particularly useful to reduce hepatic inflammation and could be a pharmacologically useful agent for human NASH, but probably in combination with other agents.

## Introduction

Non-alcoholic fatty liver disease (NAFLD) is closely associated with obesity, insulin resistance and hyperlipidemia and is considered as the hepatic manifestation of the metabolic syndrome. NAFLD is defined by the accumulation of fat in the liver, in the absence of excessive alcohol consumption. A more severe form of NAFLD is non-alcoholic steatohepatitis (NASH), characterized by steatosis in concert with inflammation, which can lead to liver fibrosis and cirrhosis. Current management is primarily focused on promoting weight loss through lifestyle interventions. Although NASH has emerged as a rising and major form of chronic liver disease worldwide, there is still no approved pharmacotherapy for NASH. Therefore a tremendous effort is put worldwide in the development of novel NASH therapeutics^1^. This development requires the use of animal models that adequately mimic the human disease. However, several preclinical models do not have the same metabolic syndrome-like context seen in most NASH patients or do not represent the underlying disease pathways^2,3^. Since many novel NASH therapeutics have failed in clinical trials^4^, this has raised questions on the adequacy of preclinical models.

Elafibranor is one of the drugs that showed beneficial effects in different animal models^5^ and was lately tested in patients with NASH and fibrosis in a clinical phase 3 trial. Elafibranor is a dual agonist acting upon the peroxisome proliferator-activated receptors (PPARs) α and δ, nuclear receptors that play a key role in cellular processes regulating lipid metabolism and fatty acid transport and oxidation, but affect glucose metabolism and inflammation as well^6–9^. Elafibranor revealed beneficial effects in NASH patients during a phase 2 trial^10^, but interim results of the ongoing RESOLVE-IT phase 3 trial with monotreatment of elafibranor have reported a failure to demonstrate a significant effect on NASH resolution^11^. However, the RESOLVE-IT study will be continued and combination therapies of elafibranor with other NASH therapeutics are still being launched^12^.

In the current study we investigated the response of elafibranor in APOE*3Leiden.human Cholesteryl Ester Transfer Protein (E3L.CETP) mice. E3L.CETP mice are a well-established model for hyperlipidemia and atherosclerosis^13,14^. When fed a high fat diet the mice display characteristics of the metabolic syndrome^15^ and with cholesterol supplementation they develop NASH in the context of obesity, insulin resistance and hyperlipidemia^16–19^. The model has been proven to respond to several hypolipidemic and anti-diabetic drugs similarly as in humans^20–27^. Using this translational model, we evaluated the response of elafibranor on plasma parameters and NASH histopathology, and hepatic transcriptome analysis was used to investigate the underlying pathways affected by elafibranor. The underlying disease pathways of the model were compared with those of NASH patients and we discuss the response of elafibranor in the mice as compared to the response in human patients, as well as potential pitfalls when translating preclinical results of novel NASH therapeutics to human patients.

## Methods

### Animals and experimental design

All animal care and experimental procedures were approved by the Ethical Committee on Animal Care and Experimentation (Zeist, The Netherlands), and were in compliance with European Community specifications regarding the use of laboratory animals. The study was carried out in compliance with ARRIVE guidelines. Homozygous human cholesteryl ester transfer protein (CETP) transgenic mice (strain 5203)^15,28^ were obtained from Jackson Laboratories (Bar Harbor, ME, USA) and cross-bred with E3L mice^29^ in our local animal facility at TNO to obtain heterozygous E3L.CETP mice^14,30,31^. Mice were group housed in a temperature-controlled room on a 12 h light–dark cycle and had free access to food and heat sterilized water. For the experiment 20–22 week old male APOE*3Leiden.CETP mice were matched on age, body weight, blood glucose and plasma cholesterol and triglycerides into one age-matched healthy reference group of 8 mice that were kept on the healthy chow diet (R/M-H, Ssniff Spezialdieten GmbH, Soest, Germany) and a group of 36 mice that were given a high fat and cholesterol diet (HFC) containing 45 kcal% fat derived from lard (Cat. no. 12451), supplemented with 1% (w/w) cholesterol (Research Diets, New Brunswick, NJ, USA) for 15 weeks to induce NASH. After 15 weeks mice on the HFC diet were matched on age, body weight, blood glucose and plasma cholesterol and triglycerides into one group that was left untreated (HFC control group, n = 15) and one group of mice that were treated with the PPAR-α/δ agonist elafibranor (Bio-Connect, Huissen, The Netherlands) provided as diet admix (15 mg/kg/d) from week 15–25 (n = 15). In addition, a small (n = 6) HFC reference group was added that was sacrificed at t = 15 weeks to indicate the severity of NASH and fibrosis at the start of the treatment. Comparison of the elafibranor treated group with this small reference group led to an indication whether elafbibranor treatment could improve certain NASH/fibrosis characteristics beyond the levels at the start of the treatment or merely blocked the further progression. Animals were sacrificed unfasted by gradual-fill CO_2_ asphyxiation in week 15 (HFC reference group) or week 25 (other groups). Body weight and food intake per cage were measured regularly during the study (at t = 0, 15, 20 and 25 weeks). Blood samples were taken from the tail vein after 4 h fasting (with blood withdrawn around 08.00 h) in EDTA coated tubes (Sarstedt, Nümbrecht, Germany). Terminal blood was collected through cardiac heart puncture to prepare EDTA plasma and livers and perigonadal, visceral and subcutaneous white adipose tissue (WAT), were collected, weighed and fixed in formalin and paraffin-embedded (lobus sinister medialis hepatis and lobus dexter medialis hepatis) for histological analysis or (remaining liver lobes) fresh-frozen in N_2_ and subsequently stored at -80 °C for biochemical analysis and gene expression analysis.

### Plasma and liver biochemical analysis

Blood glucose was measured at the time of blood sampling using a hand-held glucometer (Freestyle Disectronic, Vianen, The Netherlands). Plasma insulin was analysed by ELISA (Mercodia AB, Uppsala, Sweden). Plasma cholesterol and triglycerides were determined using enzymatic assays (CHOD-PAP and GPO-PAP, respectively; Roche Diagnostics, Almere, The Netherlands). HDL-cholesterol was also quantified for each mouse individually in plasma after precipitation of apoB-containing lipoproteins using PEG/glycine, as previously described^24^. The distribution of cholesterol of the various lipoproteins was determined in plasma pooled per group after separation of lipoproteins by fast-performance liquid chromatography (FPLC) using a Superose 6 column^26^. Plasma alanine aminotransferase (ALT) and aspartate aminotransferase (AST) were measured using a spectrophotometric activity assay (Reflotron-Plus, Roche). Hepatic collagen content was measured via a hydroxyproline based colorimetric assay as marker of fibrosis using the Sensitive total collagen assay (Quickzyme, Leiden, The Netherlands). Intrahepatic concentration of triglycerides, free cholesterol and cholesteryl esters was determined as described previously^32^. Briefly, approximately 50 mg of tissue was homogenized in phosphate buffered saline and samples were taken for measurement of protein content. Lipids were extracted and separated by high performance thin layer chromatography (HPTLC) on silica gel plates. Lipid spots were stained with colour reagent (5 g MnCl2̣.4H2O, 32 mL 95–97% H2SO4 added to 960 mL of CH3OH:H2O 1:1 v/v) and quantified using Image Lab software (version 5.2.1, Bio-Rad Laboratories B.V., Veenendaal, The Netherlands).

### Histology

Liver samples (lobus sinister medialis hepatis and lobus dexter medialis hepatis) were collected (from non-fasted mice), fixed in formalin and paraffin embedded, and 3 µm sections were stained with hematoxylin and eosin (H&E) and Sirius Red. NASH was scored blindly by a board-certified pathologist in H&E stained cross sections using an adapted grading system of human NASH^33,34^. In short, the level of macrovesicular and microvesicular steatosis was determined (in two separate cross-sections of medial lobe mounted on one glass) at 40× to 100× magnification relative to the total liver area analysed and expressed as a percentage. Inflammation was scored by counting the number of aggregates of inflammatory cells per field using a 100× magnification (view size of 4.2 mm^2^). The average of five random fields were taken within those two cross-sections and values were expressed per mm^2^. Hepatic fibrosis was identified using Sirius Red stained slides and evaluated as well using two cross-sections by computerized image analysis of hepatic collagen content (as percentage of liver surface area and including blood vessels). In addition a qualitative analysis regarding the fibrosis stage was performed by a certified pathologist using the protocol of Tiniakos et al.^35^, in which the presence of pathological collagen staining was scored within two cross-sections of medial lobe as either absent (F0), observed within perisinusoidal/perivenular or periportal area (F1), within both perisinusoidal and periportal areas (F2), bridging fibrosis (F3) or cirrhosis (F4).

### Transcriptome analysis

Nucleic acid extraction was performed as described previously in detail^36^. Total RNA was extracted from individual lobus dexter lateralis samples using glass beads and RNA-Bee (Campro Scientific, Veenendaal, The Netherlands). RNA integrity was examined using the RNA 6000 Nano Lab-on-a-Chip kit and a bioanalyzer 2100 (Agilent Technologies, Amstelveen, The Netherlands). The NEBNext Ultra II Directional RNA Library Prep Kit (NEB #E7760S/L, New England Biolabs, Ipswich, MA, USA) was used to process the samples. Briefly, mRNA was isolated from total RNA using the oligo-dT magnetic beads. After fragmentation of the mRNA, cDNA synthesis was performed, cDNA was ligated with the sequencing adapters and amplified by PCR. Quality and yield of the amplicon was measured (Fragment Analyzer, Agilent Technologies, Amstelveen, The Netherlands ) and was as expected (broad peak between 300 and 500 bp) and a concentration of 1.1 nM of amplicon-library DNA was used. Clustering and DNA sequencing, using the Illumina NovaSeq6000, was performed according to manufacturer's protocols by service provider GenomeScan B.V (Leiden, the Netherlands), yielding 15–30 million sequencing clusters per sample and 2 × 150 bp Paired-End reads (PE) per cluster. The genome reference and annotation file Mus_musculus.GRCm38.gencode.vM19 was used for analysis in FastA and GTF format. The reads were aligned to the reference sequence using the STAR 2.5 algorithm with default settings (https://github.com/alexdobin/STAR). Based on the mapped read locations and the gene annotation HTSeq-count version 0.6.1p1 was used to count how often a read was mapped on the transcript region. These counts serve as input for the statistical analysis using DEseq2 package^37^. Selected differentially expressed genes (DEGs), corrected for multiple testing, were used as an input for pathway analysis (*P* value < 0.000001) through Ingenuity Pathway Analysis suite (www.ingenuity.com, accessed 2020).

To evaluate the representation of human pathophysiological pathways in HFC-fed E3L.CETP mice, murine hepatic gene expression profiles were compared with published data on hepatic gene expression profiles in human NASH patients versus control. To this end, hepatic gene expression of NASH patients and controls of four different human studies from the Gene Expression Omnibus (GEO) with accession numbers GSE48452, GSE61260, GSE89632 and GSE33814^38–41^ were used. A unique gene symbol list over all studies was used to identify common expression results over the various studies and 2logR and P-values were calculated using NCBI GEO2R (https://www.ncbi.nlm.nih.gov/geo/geo2r/?acc=GSE48452 or GSE89632 or GSE33814). For study GSE61260 normalised counts-data were used to calculate P-vales and 2logR. Only the differentially expressed genes that were found in at least two studies AND had the same 2logR direction were used as an input for pathway analysis (*P* values < 0.01) through Ingenuity Pathway Analysis suite (www.ingenuity.com, accessed 2020). In addition, the representation in E3L.CETP mice of human pathophysiological pathways specific for severe fibrosis, was evaluated by comparing the murine gene expression with published data of a study that differentiates NASH patients with severe fibrosis (fibrosis stage F3 or 4) from NASH patients with mild fibrosis (fibrosis stage F0 or 1) (GEO accession number GSE31803)^42^.

### Statistical analysis

All values shown represent means ± SEM. Statistical differences between groups were determined by using non-parametric Kruskal–Wallis followed by Mann–Whitney U test for independent samples using SPSS software. A *P* value < 0.05 was considered statistically significant. Two-tailed *p* values were used. In the case of transcriptome analysis we selected differentially expressed genes using p-values, adjusted for multiple testing (False Discovery Rate, FDR) < 0.01 AND abs2logRatio > 0.5. The differentially expressed pathways (DEP) were selected based on Fischer’s exact test in the Ingenuity Pathway Analysis Software.

## Results

### Elafibranor reduces features of the Metabolic Syndrome in E3L.CETP mice

**Figure 1:**
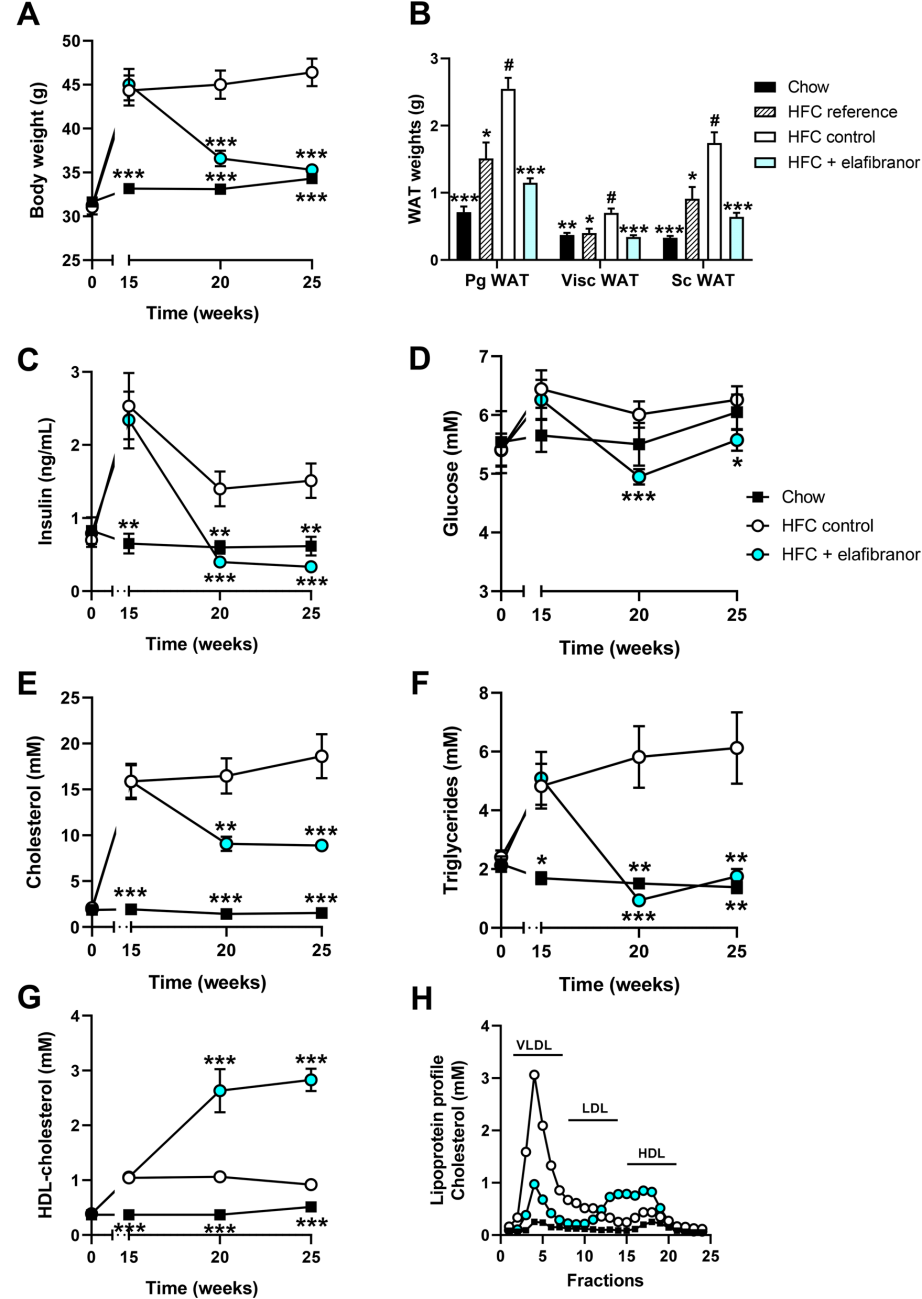

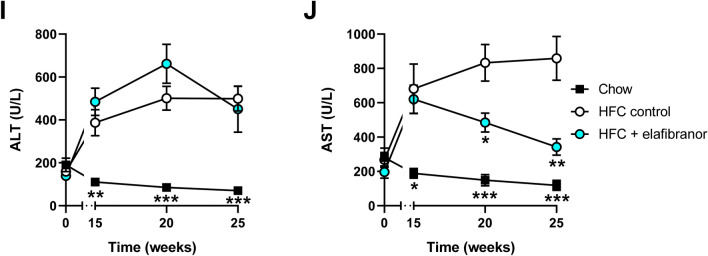
Metabolic parameters in E3L.CETP mice improved by elafibranor. Body weight (**A**), WAT weight of different adipose tissue depots (**B**), plasma insulin (**C**), blood glucose (**D**), plasma cholesterol (**E**), plasma triglycerides (**F**), plasma HDL-cholesterol (**G**), lipoprotein profiles in plasma samples pooled per group (**H**), plasma ALT (**I**) and plasma AST (**J**) from E3L.CETP mice fed a healthy chow diet or fed a HFC diet for 15 weeks and left untreated or treated with 15 mg/kg/d elafibranor for 10 weeks. Values represent mean ± SEM for n = 8 chow mice, n = 6 HFC reference mice and n = 15 HFC control or HFC + elafibranor treated mice. * *p* < 0.05, **p < 0.01 and ***p < 0.001 *vs.* HFC control; #*p* < 0.05 *vs.* HFC reference.

E3L.CETP mice fed the HFC diet developed pronounced obesity (as compared to age-matched control mice fed a low fat chow diet) within 15 weeks that remained stable until 25 weeks (Fig. 1A). Treatment with elafibranor resulted in a significant lowering of body weight (with −24%, *p* < 0.001 at t = 25) as compared to the HFC control group (Fig. 1A), despite receiving the same HFC diet and food intake being similar or slightly higher during the study in the elafibranor group (data not shown: 3.2 ± 0.1 g/mouse/day *vs.* 2.9 ± 0.2 g/mouse/day, *p* = 0.126, respectively; average food intake values of ≥ 5 cages at t = 20 and t = 25 weeks). The HFC diet resulted in a gradual increase in perigonadal, visceral and subcutaneous WAT weights after 15 weeks (HFC reference group) and 25 weeks (HFC control group), while treatment with elafibranor resulted in significantly lower WAT weights as compared to the HFC control group (with −55%, −52% and −63%, all *p* < 0.001 for perigonadal, visceral and subcutaneous WAT, respectively; Fig. 1B). Plasma insulin levels significantly increased on the HFC diet after 15 weeks and then decreased again at t = 20 to remain at a stable hyperinsulinemic level until t = 25 weeks, while glucose levels remained similar to the chow fed animals (Fig. 1C, D). Elafibranor treatment resulted in a significant decrease in both insulin and glucose levels as compared to the HFC control group (insulin with -71% and -78%, both *p* < 0.001, at t = 20 and t = 25, respectively; glucose with −18%, *p* < 0.01 and −11%, *p* = 0.026 at t = 20 and 25, respectively; Fig. 1C, D).

In response to the HFC diet, mice developed stable hypercholesterolemia and severe hypertriglyceridemia (cholesterol: 11.6 and 12.2-fold increase, both *p* < 0.001, *vs.* chow diet at t = 20 and t = 25, respectively; triglycerides: 3.9-fold, *p* = 0.001 and 4.4-fold, *p* = 0.013, increase *vs.* chow diet for t = 20 and t = 25, respectively) (Fig. 1E, F). The increase in cholesterol was primarily due to an increase in very low-density lipoprotein (VLDL), although low-density lipoprotein (LDL) and high-density lipoprotein (HDL) cholesterol were increased as well (Fig. 1G, H). Elafibranor treatment significantly lowered plasma cholesterol and triglyceride levels as compared to the HFC control group (cholesterol with −45%, *p* = 0.009 and −52%, *p* = 0.001, at t = 20 and t = 25, respectively; triglycerides with −84%, *p* < 0.001 and −71%, *p* = 0.011, at t = 20 and t = 25, respectively; Fig. 1E, F). The decrease in cholesterol with elafibranor was primarily due to a reduction in VLDL and LDL, while HDL-cholesterol was significantly increased and a larger cholesterol ester (CE)- and apoE-rich HDL-particle^27,43^ was formed (Fig. 1G, H).

The HFC diet led to an increased liver weight as compared to the chow diet (data not shown: 3.7 ± 0.3 g *vs.* 1.7 ± 0.1 g, *p* < 0.001) and analysis of liver enzymes showed a concomitant increase in plasma ALT (5.9-fold, *p* = 0.005 and 7.1-fold, *p* < 0.001, at t = 20 and t = 25, respectively) and AST (5.6-fold, *p* = 0.035 and 7.2-fold, *p* < 0.001, at t = 20 and t = 25, respectively) as compared to the chow diet, indicating that the HFC diet caused liver damage (Fig. 1I, J). Elafibranor treatment increased liver weight even further, typical for a compound with PPARα-agonistic activity (to 5.2 ± 0.1 g, *p* < 0.001 both *vs.* chow and HFC control), and did not significantly affect plasma ALT levels, while plasma AST levels were significantly decreased (with −42%, *p* = 0.029 and −60%, *p* = 0.004, at t = 20 and t = 25, respectively) as compared to the HFC control group (Fig. 1I, J).

### Elafibranor reduces steatosis and hepatic inflammation and blocks progression of fibrosis in E3L.CETP mice

**Figure 2:**
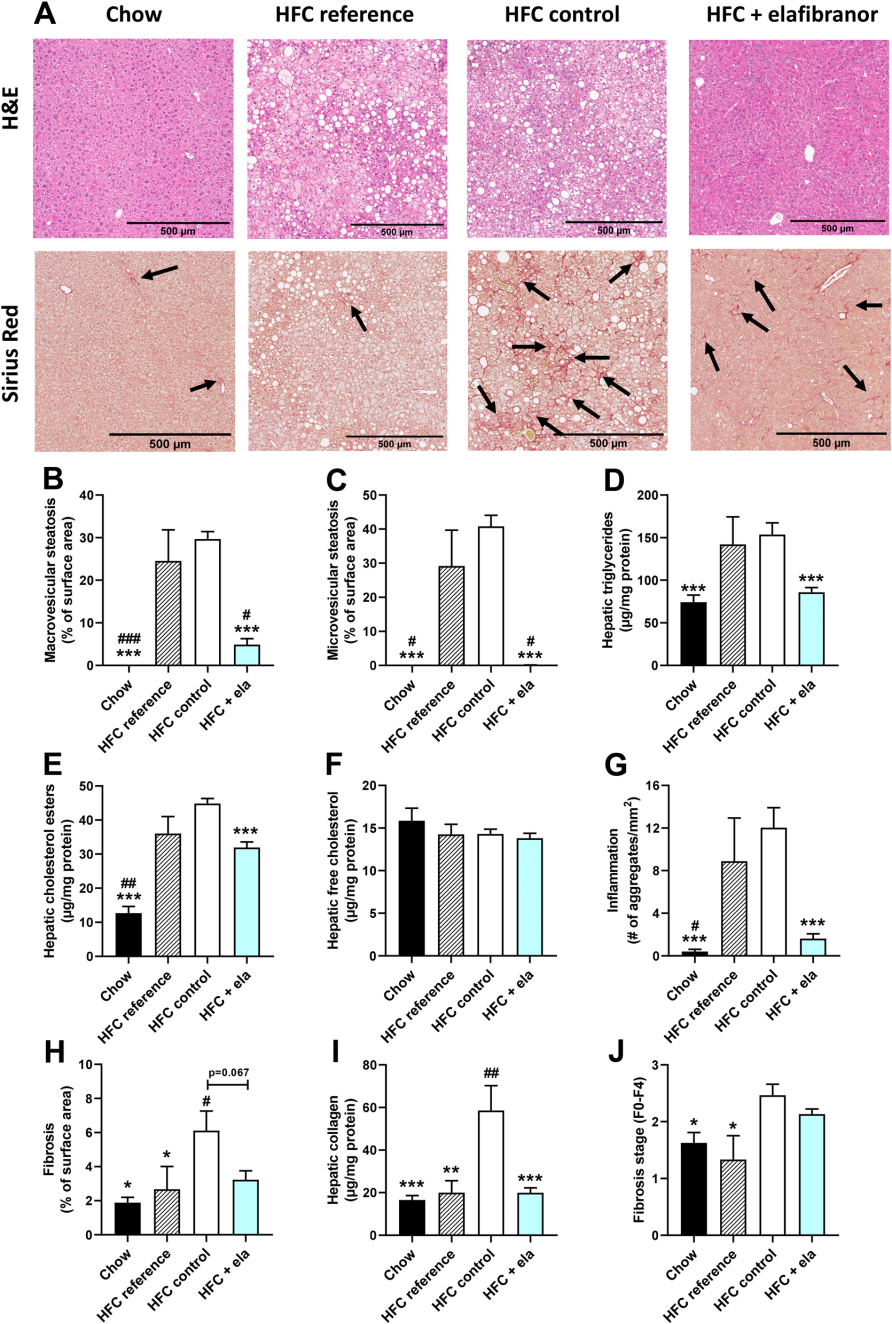
Histological photomicrographs and quantitative analysis of NASH in E3L.CETP mice. Representative images of liver cross sections stained with hematoxylin and eosin (H&E) or Sirius Red (arrows indicate collagen depositions) **(A)** and quantitative analysis (**B**–**I**) from E3L.CETP mice fed a healthy chow diet or fed a HFC diet for 15 weeks and left untreated or treated with 15 mg/kg/d elafibranor (ela) for 10 weeks. Macrovesicular **(B)** and microvesicular **(C)** steatosis as percentage of total liver area, biochemically analysed hepatic triglycerides (**D**), cholesterol esters (**E**) and free cholesterol (**F**), inflammation as number of inflammatory aggregates per mm^2^ microscopic field (**G**), hepatic fibrosis as percentage Sirius Red of surface area (**H**) or biochemically analysed hepatic collagen (**I**) and hepatic fibrosis stage (**J**). Values represent mean ± SEM for n = 8 chow mice, n = 6 HFC reference mice and n = 15 HFC control or HFC + elafibranor treated mice. *p < 0.05, **p < 0.01 and ***p < 0.001 *vs.* HFC control; ^#^*p* < 0.05, ^##^*p* < 0.01 and ^###^*p* < 0.001 *vs.* HFC reference.

HFC feeding induced pronounced steatosis after 15 weeks (HFC reference group) and 25 weeks (HFC control group) of diet feeding, while treatment with elafibranor decreased this beyond the levels at the start of the treatment at 15 weeks (Fig. 2A). Quantitative analysis (Fig. 2B, C) revealed that after 15 weeks about 54% of the surface area was steatotic, of which 25% consisted of macrovesicular steatosis and 29% of microvesicular steatosis. After 25 weeks about 71% of the surface area was steatotic, of which 30% consisted of macrovesicular steatosis and 41% of microvesicular steatosis. Treatment with elafibranor fully blunted the microvesicular steatosis to 0.1% and only a slight macrovesicular steatosis of 5% remained. Biochemical analysis of intrahepatic liver lipids (Fig. 2D, E and F) was in line with the histological analysis and revealed that HFC feeding resulted in a significant increase in hepatic triglycerides and cholesterol esters as compared to the chow diet (2.1-fold and 3.5-fold increase after 25 weeks, both *p* < 0.001), while free cholesterol levels remained similar. Elafibranor treatment almost normalized the hepatic triglyceride levels (−44% decrease *vs.* HFC control, *p* < 0.001) and significantly decreased hepatic cholesterol esters as well (−29% decrease *vs.* HFC control, *p* < 0.001), while free cholesterol levels remained unchanged.

HFC feeding also strongly induced lobular inflammation, characterized by aggregates of inflammatory cells comprising mononuclear cells and polymorphonuclear cells. Quantification of the lobular inflammation (Fig. 2G) showed that the HFC feeding resulted in a robust increase in the number of aggregates as compared to the chow diet (20.5-fold increase, *p* = 0.029 and 27.8-fold increase, *p* < 0.001, after 15 weeks and after 25 weeks, respectively). Treatment with elafibranor largely decreased the number of inflammatory aggregates (7.4-fold decrease *vs.* HFC control, *p* < 0.001).

Fifteen weeks of HFC feeding induced onset of fibrosis, as shown by the patches of collagen deposition and after 25 weeks the fibrosis was evidently existing (Fig. 2A; Sirius Red staining). Quantification of fibrosis by computerized analysis of collagen deposition in histological slices (Fig. 2H) revealed a profound increase in collagen deposition after 25 weeks of HFC feeding (3.3-fold increase *vs.* chow diet, *p* = 0.011) that was confirmed by biochemical analysis of collagen (Fig. 2I; 3.5-fold increase *vs.* chow diet, *p* < 0.001). Treatment with elafibranor precluded this induction of fibrosis, as shown by the significant lower levels as compared to the HFC control group in biochemical analysis (2.9-fold, *p* < 0.001, respectively) and tendency with histological collagen content (1.9-fold, *p* = 0.067). Fibrosis evaluation by a board-certified pathologist revealed that after 15 weeks of HFC feeding for most mice fibrosis was primarily located within perisinusoidal and/or periportal area (score F1–F2) and after 25 weeks of HFC feeding the majority (60%) of mice revealed bridging fibrosis (F3) (and the remaining mice had fibrosis in perisinusoidal and/or periportal area, score F1–F2)(Fig. 2J). Almost all mice (87%) with elafibranor treatment had fibrosis within perisinusoidal and periportal area (score F2) and the remaining mice revealed bridging fibrosis (F3) (Fig. 2J).

### Treatment with elafibranor normalizes metabolic, inflammatory and fibrotic gene expression

**Figure 3:**
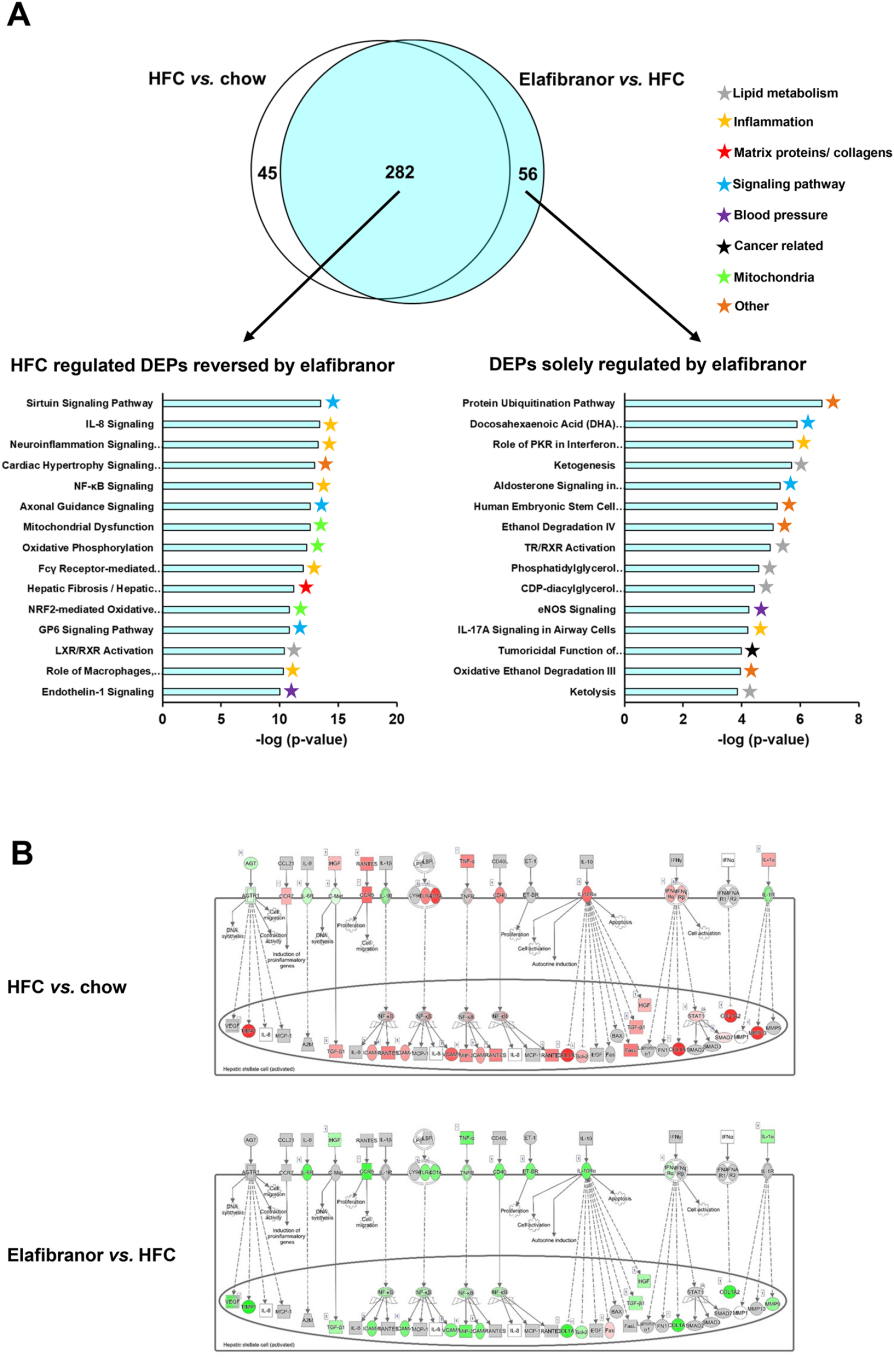

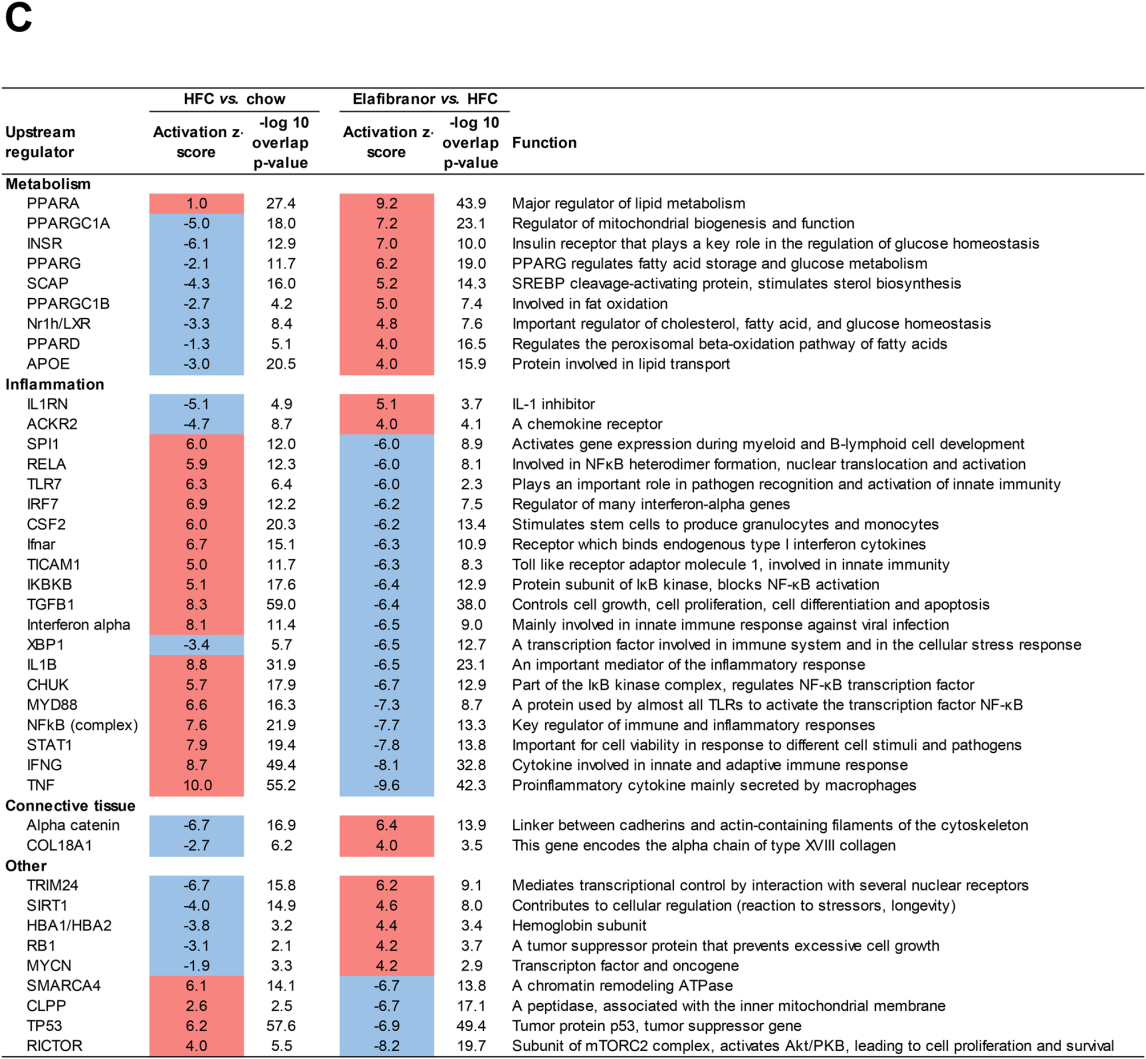
Elafibranor normalizes differentially expressed pathways and upstream regulators in E3L.CETP mice induced by HFC diet. Venn diagram (**A**) illustrating the overlap of differentially expressed pathways (pathway *p*-val < 0.01) of livers between E3L.CETP mice fed a HFC diet *vs.* healthy chow diet for 25 weeks (white circle) and E3L.CETP mice on HFC diet and treated with elafibranor *vs.* untreated mice (blue circle). The top 15 of significantly enriched pathways (−log(*P*-value)) for the genes affected by HFC diet and reversed by elafibranor, as well as for the genes affected by elafibranor but not induced by HFC are shown. Pathway analysis (**B**) showing significant gene expression changes in activated hepatic stellate cells in E3L.CETP mice on HFC diet *vs.* chow (upper panel) and E3L.CETP mice on HFC diet treated with elafibranor *vs.* untreated mice (lower panel). Red colour indicates upregulation and green colour indicates down-regulation. Upstream regulator analysis (**C**) showing the predicted activation state (z-score) of the upstream regulators, based on the expression changes of known target genes. The overlap *p*-value indicates the significance of the overlap between the known target genes of a transcription factor and the differentially expressed genes measured in the experiment. Red colour indicates upregulation and blue colour indicates down-regulation. Ranking is based on the largest upregulation to the largest downregulation of upstream regulators induced by elafibranor as indicated by the z-score.

To further investigate the mechanisms and pathways modulated by elafibranor, differentially expressed pathways of E3L.CETP mice treated with elafibranor were compared with untreated mice. While HFC diet led to a total of 327 differentially expressed pathways as compared to the mice on a healthy chow diet, elafibranor treatment resulted in a total of 338 differentially expressed pathways, the majority of which (83%) overlapped with the differentially expressed pathways that were induced by HFC diet (Fig. 3A). The far majority of those overlapping pathways were also reversed by elafibranor treatment and only a small portion of the pathways were attenuated or enhanced by elafibranor treatment per se. The top 15 most significantly enriched pathways for the overlapping part of the Venn diagram that were induced by HFC diet and reversed by elafibranor treatment, as well as the top 15 most significantly enriched pathways that were not induced by HFC diet but only affected by elafibranor treatment, are visualized in Fig. 3A. Among the pathways that were reversed by elafibranor treatment were important pathways for NASH development, like inflammation pathways such as NF-κB and IL-8 signalling and metabolism pathways (sirtuin signalling, mitochondrial function and oxidative phosphorylation, LXR/RXR activation), as well as hepatic fibrosis/hepatic stellate cell activation. Part of the hepatic fibrosis pathway analysis representing the statistically significant gene expression changes in activated stellate cells is shown in Fig. 3B and indicates an upregulation for most genes with HFC diet and a reversal of this gene expression upon elafibranor treatment.

We subsequently performed an upstream regulator analysis as well that determines the activation state (z-score) of the transcription factors involved, based on the changes in expression of their target genes. All significant upstream regulators with a z-score < -6 and > 4 (arbitrary cut-offs to shorten the list) for elafibranor *vs.* HFC are shown in Fig. 3C. The majority of the upstream regulators belonged to biological process categories with high relevance to NASH development, like ‘Metabolism’, ‘Inflammation’ and ‘Connective tissue’. Elafibranor led to inhibition of inflammatory upstream regulators, and as expected from a PPAR-α/δ agonist to upregulation of PPAR-α and PPAR-δ, as well as PPAR-γ and other metabolic upstream regulators.

### E3L.CETP mice on HFC diet have a transcriptomic profile similar to humans with NASH

**Figure 4:**
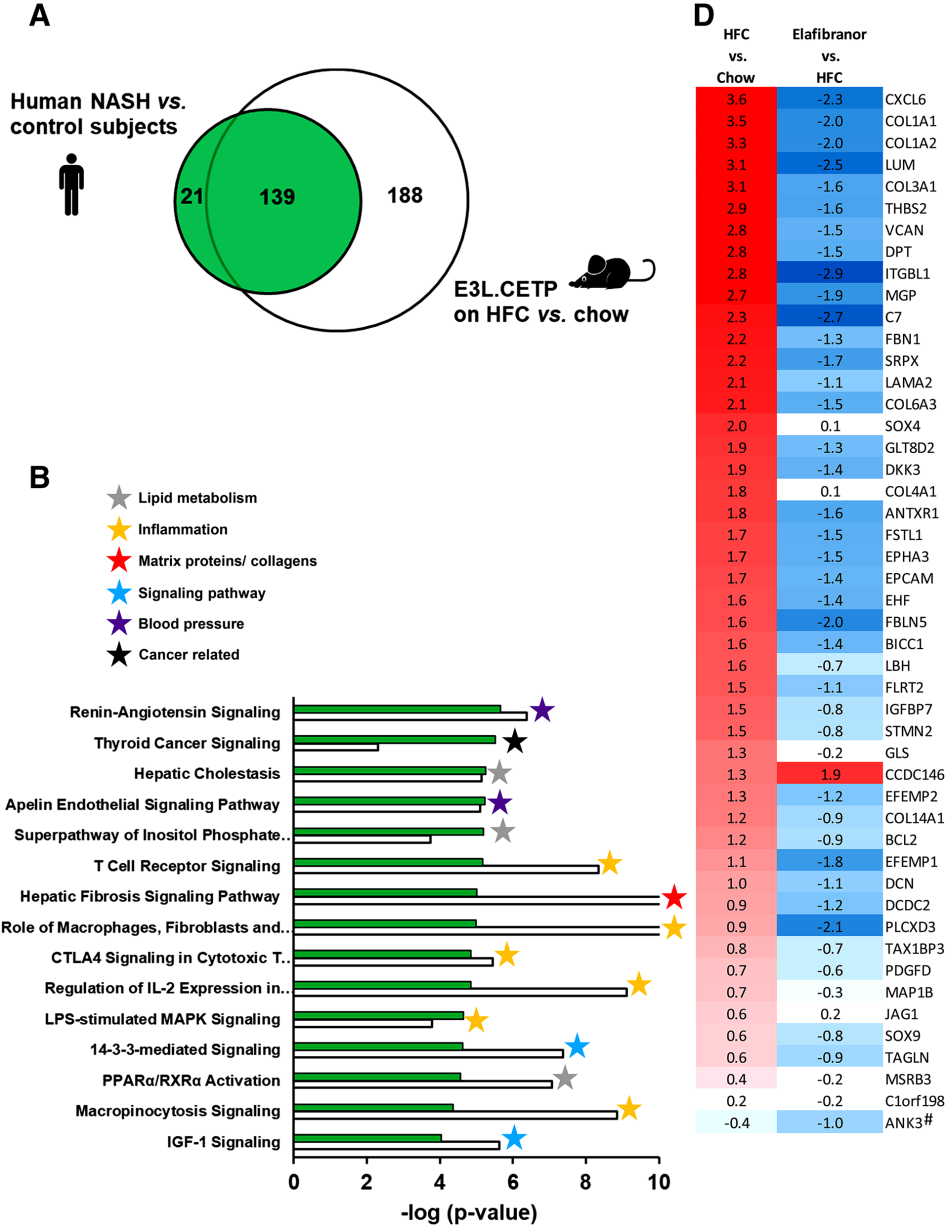

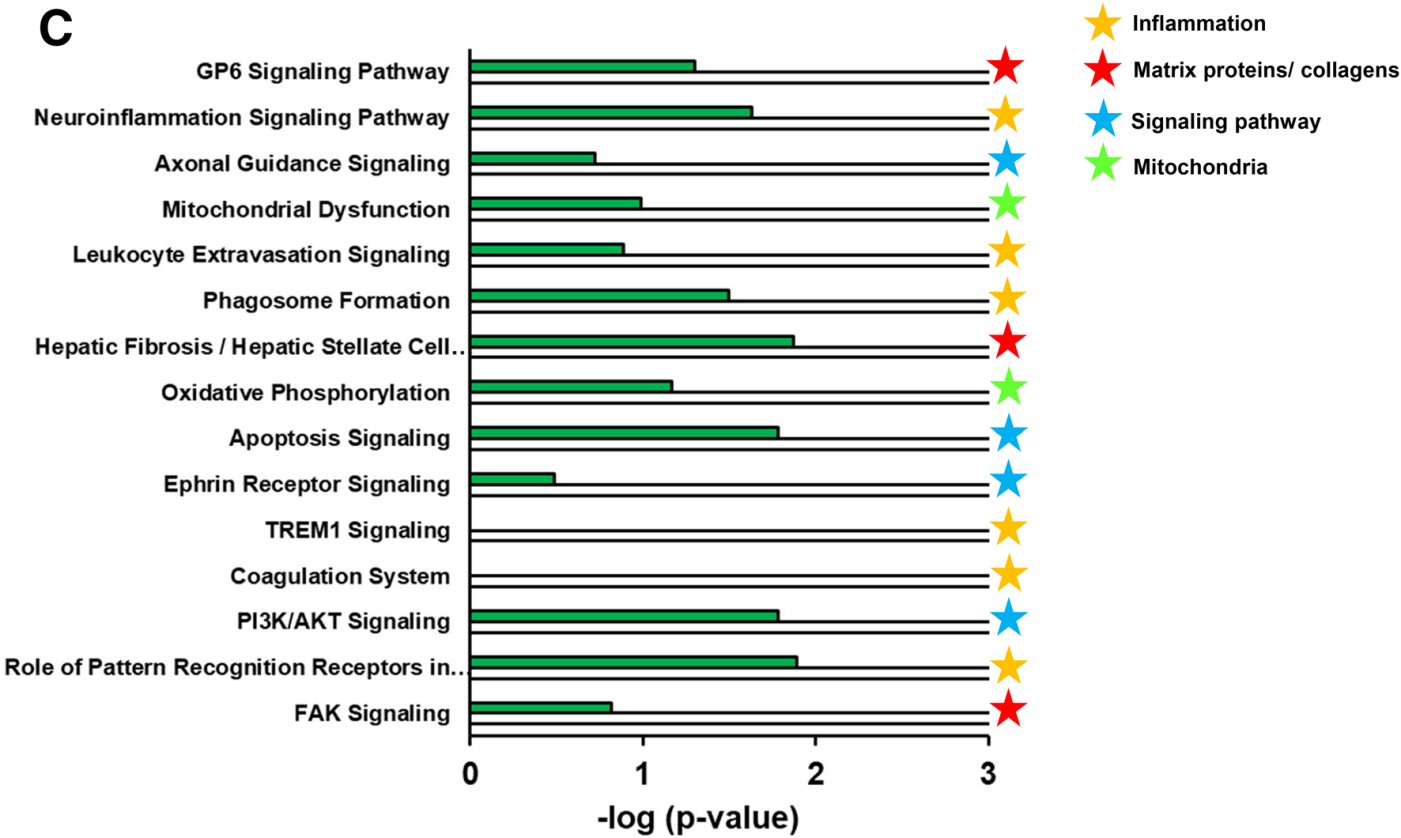
Differentially expressed pathways in E3L.CETP mice as compared to NASH patients. Venn diagram (**A**) illustrating the overlap of differentially expressed pathways (pathway *p*-val < 0.01) of livers between E3L.CETP mice fed a HFC diet *vs.* healthy chow diet for 25 weeks (white circle) and human NASH patients *vs.* healthy control subjects (green circle). The top 15 of significantly enriched pathways (−log(*P*-value)) for the genes affected in NASH patients (green bars) and the enrichment of E3L.CETP mice on HFC diet herein (white bars) (**B**). *P*-values equal to or smaller than −log(*P* value) of 10 are shown as 10. The top 15 of significantly enriched pathways (−log(*P*-value)) for the genes uniquely affected in E3L.CETP mice (white bars) and the (non-significant) enrichment of NASH patients plotted herein (**C**). *P*-values equal to or smaller than −log(*P* value) of 3 are shown as 3. Heatmap (**D**) showing recapitulation in E3L.CETP mice, as well as in response to elafibranor treatment, of hepatic gene expression profile that differentiates NASH patients with mild fibrosis (stage F0 or 1) from severe fibrosis (stage F3 or 4)^42^. Red colour indicates upregulation and blue colour indicates down-regulation. Gene with # indicates gene with differential regulation in mice as compared to human regulation.

On gene expression level elafibranor in the E3L.CETP mice predominantly reversed pathways that were induced by HFC. For an appropriate judgement of the translational value of the elafibranor effects in E3L.CETP, it is important to investigate to which degree the induction of NASH in E3L.CETP mice by HFC feeding mimics the human NASH patients. To investigate whether E3L.CETP mice on HFC diet indeed recapitulate the underlying disease pathways of NASH patients, hepatic gene expression of the mice was compared with a representative human NASH signature. To this end, the published hepatic gene expression profiles of four independent human studies with NASH patients and controls^38–41^ were merged in such a way that only the differentially expressed genes that were found in at least two studies AND had the same 2logR direction were used for pathway analysis. In total, 160 differentially expressed pathways were identified in humans that distinguished NASH patients from controls. Of those, 139 or 87% were recapitulated in E3L.CETP mice on HFC diet (Fig. 4A). As compared to humans, E3L.CETP mice on HFC diet *vs.* chow diet had more (n = 327) differentially expressed pathways.

The top 15 most significantly enriched pathways for human NASH patients are visualized in Fig. 4B and the enrichment of those pathways in E3L.CETP mice on HFC diet was plotted herein. The top 15 consisted for the most part of pathways related to inflammation, followed by pathways involved in lipid metabolism. Furthermore, blood pressure and cancer related pathways rank high, as well as the hepatic fibrosis signalling pathway. In the E3L.CETP mice the top 15 of human NASH pathways were all recapitulated as well. Overall the enrichment of most inflammatory pathways and the hepatic fibrosis signalling pathway was larger in the E3L.CETP mice, while the enrichment in the cancer related pathway was lower. Additionally, the E3L.CETP mice on HFC diet had more (n = 188) differentially expressed pathways that were not observed in humans. The top 15 most enriched pathways of this portion of the Venn diagram is shown in Fig. 4C and reveals predominantly pathways related to inflammation, but matrix proteins/collagens and mitochondria and signalling pathways as well. Most of those pathways were enriched in humans as well, but did not reach the -log (p-value) cut-off of 2.

In addition to the human NASH gene signature, we investigated the recapitulation of fibrosis pathways in more detail. Hereto, a published human gene profile that specifically differentiates NASH patients with severe fibrosis (stage F3 or F4) from NASH patients with mild fibrosis (stage F0 or F1) was used^42^. This differential gene set consists of genes that are all upregulated in the NASH patients with severe fibrosis. This gene set was significantly upregulated as well, for all except one gene, in the E3L.CETP mice on HFC (Fig. 4D). Treatment with elafibranor in the E3L.CETP mice predominantly reversed the gene expression of this gene set (Fig. 4D).

## Discussion

In this study, we demonstrate that treatment of obese, insulin-resistant and dyslipidemic E3L.CETP mice with elafibranor markedly ameliorated steatosis and lobular inflammation and blunted the progression of hepatic fibrosis. Bioinformatics analysis of gene expression identified regulatory pathways and upstream regulators in the liver that are specifically influenced by elafibranor.

To mimic the human situation of NASH patients as closely as possible, we used a transgenic mouse model with a lipoprotein metabolism resembling humans and used a dietary induction to represent the obesogenic diets to which many NASH patients are exposed. E3L.CETP mice, due to their genetic APOE*3Leiden mutation, have an impaired clearance of apoB-containing lipoproteins, thereby mimicking the slow clearance observed in humans and resulting in a mouse model that develops hyperlipidemia and atherosclerosis upon saturated fat and cholesterol feeding^13,14,31^. The model is therefore in clear contrast to wild-type C57BL6 mice that have a very rapid clearance of apoB-containing particles resulting in plasma cholesterol that is primarily contained in the HDL particle (and do not develop atherosclerosis). The high fat and cholesterol diet that was used in the current study induced obesity, insulin resistance and hyperlipidemia in the model and the hepatic phenotype of the mice resembled the human NASH pathology^33,34^. The model developed a substantial amount of macrovesicular steatosis, a hallmark of NASH patients, besides microvesicular steatosis. In addition, lobular (mixed) inflammation and increasing fibrosis, progressing to bridging fibrosis, developed in a pattern typical for dietary induction and resembling the human situation^34^. Another important feature of the E3L.CETP mice is that in contrast to wild-type mice they respond well to treatment with hypolipidemic drugs including statins and fibrates similarly as humans do^13,15,19–23,25–27,30,31^. There is increasing evidence that statins and fibrates may have beneficial effects on NASH and liver fibrosis^9,44,45^.

In phase 2a trials in obese and dyslipidemic or obese and prediabetic patients, 80 mg/d elafibranor consistently improved plasma lipids (decrease in plasma triglycerides and LDL-cholesterol, increase in HDL-cholesterol), improved glucose homeostasis (decrease in plasma glucose, fructosamine, insulin and HOMA-IR, improvement of hepatic and peripheral insulin resistance during hyperinsulinemic euglycemic clamps) and in addition improved the levels of liver enzymes (plasma alanine aminotransferase, alkaline phospatase and γ-glutamyltransferase)^46,47^. In the subsequent phase 2 trial in NASH patients, 80 and 120 mg/d elafibranor were evaluated after 1 year for resolution of NASH without worsening of fibrosis and demonstrated that the 120 mg/d dose (but not the 80 mg/d dose) improved NAFLD activity score (NAS) without worsening of fibrosis^10^. A phase 3 trial with 120 mg/d elafibranor in NASH patients is currently ongoing and unfortunately, interim results of this trial reported that after 72 weeks the placebo arm had an unexpectedly high response and elafibranor failed to demonstrate a significant effect on the primary endpoint of NASH resolution without fibrosis worsening (19.2% of the patients met the primary endpoint in elafibranor treated group *vs.* 14.7% in placebo treated group, *p* = 0.0659)^11^.

In the current study in E3L.CETP mice, 15 mg/kg/d elafibranor administered after induction of disease significantly decreased plasma triglycerides and total cholesterol, increased HDL-cholesterol and decreased blood glucose and plasma insulin levels, similarly as seen in the human trials. In addition, we observed in the E3L.CETP mice a profound improvement in steatosis and hepatic inflammation, while precluding fibrosis development when treatment was started. These results are in line with the improvement in NAS score (or more specifically in steatosis and lobular inflammation score) in the GOLDEN505 phase 2 trial and corroborate as well the results of other reported preclinical rodent studies^5,48^.

Although the responses to elafibranor in our study are in line with the phase 2 clinical trials and with the results of other preclinical studies, the recent interim results of the clinical phase 3 trial (that report a failure to meet the endpoint), demand for a critical view on the discrepancy between the very promising results in all kind of different preclinical models *vs.* the so far disappointing results of the phase 3 trial. In the clinical trials a dose of 120 mg/d was used. To translate this dose to the dosing used in mouse studies, the following simplified calculation can be used as a rough guide: 120 mg in a human of 80 kg would correspond with 1.5 mg/kg/d which would be equivalent to a dose of 15 mg/kg/d in mice, when taking into account the approximately 10 times faster metabolism in mice^49^. Although some preclinical studies used a relative high dose (30 mg/kg/d)^5,48^, in our study (15 mg/kg/d) and in a *db/db* mice study (1, 3 and 10 mg/kg/d)^5^ a similar or lower doses, respectively, were used, suggesting the discrepancy cannot simply be explained by the dose.

In the clinical trials a NAS score is used for evaluation of NASH improvement. The NAS score is a combined score based on scoring of steatosis, ballooning and lobular inflammation. E3L.CETP mice on HFC diet demonstrate ballooning^34^. However, the prevalence of ballooning is not the same as seen in NASH patients and similar as for other mouse models^34,48,50^ only marginal ballooning was observed. It is therefore important to realize that rodent NASH models in general, with respect to hepatocyte ballooning, do not entirely meet the histomorphological criteria and therefore scoring of ballooning in mouse models where ballooning cells are only occasionally found, can be misleading. Therefore a direct comparison with the NAS score of the clinical trials remains difficult. The more detailed results of the GOLDEN-505 phase 2 trial report the results for a subset of the patients with a basal NAS score of ≥ 4 (so excluding the patients with a basal NAS score of 3, similarly as currently has been done in the phase 3 trial) and demonstrate large and significant effects for ballooning and lobular inflammation, while there is no significant effect on steatosis and fibrosis^10^. In contrast, in our and all other preclinical studies a profound effect on steatosis is reported. A possible clue for this difference might be given by the other deviant parameters in our study, like the decrease in body weight that is not seen in humans but has been reported in other mouse studies as well^48^. While plasma AST decreased in our study, plasma ALT was not affected. In the reported clinical trials, plasma AST was not affected while plasma ALT was consistently decreased. It has been reported that the PPAR-α agonist fenofibrate decreases AST gene expression and plasma levels in mice whereas the compound increases the expression and levels in human liver cells^51^, which may have counteracted a potential beneficial effect of elafibranor in NASH patients. Also absolute liver weights were increased in our study by elafibranor (data not shown, 1.4-fold increase *vs.* HFC control, *p* < 0.001). Weight loss, hepatomegaly, hepatocyte peroxisome proliferation, as well as increased plasma ALT levels, have been consistent findings in rodent studies with PPAR-α agonists, but not in humans^5,48,52,53^. Moreover, importantly PPARα expression in rodents may be much higher as in humans^54–57^ and PPARα activation is less pronounced in human liver compared to mouse liver^58^, and this could profoundly impact lipid metabolism and inflammation as well^8,55,59^, and subsequently development of fibrosis. It is conceivable that species-dependent metabolic effects of PPAR-α agonists explain the strong effects of elafibranor in preclinical models *vs.* the more modest effect of elafibranor in the clinical trials. An apparent contradiction to this postulate is however, that in hAPOE2/PPAR-α knockout mice^5^ steatosis has been reported to improve as well, suggesting the effect on steatosis cannot fully be explained by PPAR-α activation. However elafibranor also exhibits PPAR-δ agonistic thereby inducing peripheral fatty acid oxidation and energy metabolism and having a positive effect on lipid metabolism.

In summary, we reproduced NASH development with progression to fibrosis in HFC-fed E3L.CETP mice, a model that is characterized by obesity, metabolic anomalies and histopathological features and underlying disease pathways similar to those observed in human NASH. In this model, elafibranor exerted beneficial effects on steatosis and hepatic inflammation and a preventive effect on the progression of fibrosis. Taking into account that due to species differences the response to some targets, like PPAR-α, may be overrepresented in animal models, we infer that elafibranor will be particularly useful to reduce hepatic inflammation and could be a pharmacologically useful agent, probably in combination with other agents, for human NASH.

## Abbreviations

ALT: Alanine aminotransferase
AST: Aspartate aminotransferase
DEP: Differentially expressed pathways
E3L.CETP: APOE*3Leiden.human cholesteryl ester transfer protein
FDR: False discovery rate
GEO: Gene expression omnibus
H&E: Hematoxylin and eosin
HDL: High-density lipoprotein
HFC: High fat and cholesterol
HOMA-IR: Homeostasis model assessment for insulin resistance
LDL: Low-density lipoprotein
NAFLD: Non-alcoholic fatty liver disease
NASH: Non-alcoholic steatohepatitis
PPAR: Peroxisome proliferator-activated receptor
SEM: Standard error of the mean
VLDL: Very low-density lipoprotein
WAT: White adipose tissue
